# A cost-effective and universal strategy for complete prokaryotic genomic sequencing proposed by computer simulation

**DOI:** 10.1186/1756-0500-5-80

**Published:** 2012-01-31

**Authors:** Jingwei Jiang, Jun Li, Hoi Shan Kwan, Chun Hang Au, Patrick Tik Wan Law, Lei Li, Kai Man Kam, Julia Mei Lun Ling, Frederick C Leung

**Affiliations:** 1 School of Biological Sciences, Faculty of Science, The University of Hong Kong, Hong Kong, China; 2 Biology Programme, School of Life Sciences, The Chinese University of Hong Kong, Hong Kong SAR, China; 3 Food and Nutritional Sciences Programme, School of Life Sciences, The Chinese University of Hong Kong, Hong Kong SAR, China; 4 Food Research Centre, The Chinese University of Hong Kong, Hong Kong SAR, China; 5 Core Facilities - Genome Sequencing Laboratory, The Chinese University of Hong Kong, Hong Kong SAR, China; 6 Microbiology Division, Public Health Laboratory Services Branch, Centre for Health Protection, Department of Health, Hong Kong SAR, China; 7 Department of Microbiology, The Chinese University of Hong Kong, Prince of Wales Hospital, Hong Kong SAR, China

## Abstract

**Background:**

Pyrosequencing techniques allow scientists to perform prokaryotic genome sequencing to achieve the draft genomic sequences within a few days. However, the assemblies with shotgun sequencing are usually composed of hundreds of contigs. A further multiplex PCR procedure is needed to fill all the gaps and link contigs into complete chromosomal sequence, which is the basis for prokaryotic comparative genomic studies. In this article, we study various pyrosequencing strategies by simulated assembling from 100 prokaryotic genomes.

**Findings:**

Simulation study shows that a single end 454 Jr. run combined with a paired end 454 Jr. run (8 kb library) can produce: 1) ~90% of 100 assemblies with < 10 scaffolds and ~95% of 100 assemblies with < 150 contigs; 2) average contig N50 size is over 331 kb; 3) average single base accuracy is > 99.99%; 4) average false gene duplication rate is < 0.7%; 5) average false gene loss rate is < 0.4%.

**Conclusions:**

A single end 454 Jr. run combined with a paired end 454 Jr. run (8 kb library) is a cost-effective way for prokaryotic whole genome sequencing. This strategy provides solution to produce high quality draft assemblies for most of prokaryotic organisms within days. Due to the small number of assembled scaffolds, the following multiplex PCR procedure (for gap filling) would be easy. As a result, large scale prokaryotic whole genome sequencing projects may be finished within weeks.

## Findings

There are two major classes of prokaryotic organisms, bacteria and archaea, which genomic DNA is usually circular with genome sizes ranging from ~100 kbp to 10 Mbp. Most of these organisms cannot be cultured in the laboratory condition. They are evolving so fast, with high mutation rate and genetic drift as major evolutionary mechanisms suggested by Mira et al. [1]. Besides genetic drifts and mutations, there are other evolutionary mechanisms creating new genes in the prokaryotic genomes, including lateral gene transfer and gene duplication. Lateral gene transfer is very common among bacteria, even among the distantly related species (for example, it was thought to be the major cause of drug resistance [2]), while protein families seem to be obtained by gene duplication [3] whose duplication rate may be related to the size of the prokaryotic genome [4]. Furthermore, large-scale genomic rearrangement is another form of the prokaryotic genomic evolution and tends to frequently happen in most genomes of free-living bacteria [5].

Till recently (July, 2011), only 1673 complete microbial genomes are available in NCBI Genbank http://www.ncbi.nlm.nih.gov/genomes/lproks.cgi. Compared with large number of species of the whole prokaryotic kingdom (e.g. 350-1,500 OTUs (Operational taxonomic units) in arable or metal-polluted soils [6] and up to 500,000 OTUs in unperturbed soils [7], so large numbers that nobody is confident about how many prokaryotic species are there in nature), 1673 complete microbial genomes probably represent less than 0.1% of all prokaryotic genomes. More OTUs implies that more prokaryotic species are present in the environmental samples. In other words, much more microbial genomes await whole genome sequencing. Based on high quality draft genomes, scientists could carry out systematic evolutionary studies and comparative genomics in terms of mutations, insertions/deletions (indels), gene duplication/loss. However, the analysis of large-scale genomic rearrangement or structure variation cannot be done with draft prokaryotic genomes, but only based on complete genomes. The concept of "Pan-genome" [8] was proposed as that the genome of the prokaryotic species can be divided into "core-genome" (genes shared by all the strains) and "dispensable genome" (genes are shared either by two more strains or by a unique strain). Sequencing different strains of a prokaryotic species may provide understanding to the pan-genome of this prokaryotic species than single strain. Taken all together, the complete sequence of the prokaryotic genome is the key to a better understanding the genome evolution of these organisms.

Since 2005, different second-generation sequencing technologies (Roche/454, ABI/SOLID and Illumina/Solexa) were applied in large-scale genomic sequencing projects. These different pyrosequencing sequencing technologies are characterized by their individual features in previous study [9]. ABI/SOLID and Illumina/Solexa can produce per week ~ 250 billion bases with ~ 40-100 bp single or paired end short reads, which could cause problems in assembling the repeated regions. Roche/454 can generate much longer reads (1,000,000-1,500,000 reads ~ 400 bp in length by 454 FLX Titanium platform), but its total number of reads is far less than those generated by ABI/SOLID (400,000,000 reads ~ 50 bp in length by ABI/SOLID platform) and Illumina/Solexa (200,000,000-250,000,000 reads ~ 150 bp in length by Illumina/Solexa platform). In recent 2 years, Roche/454 has been applied in sequencing whole genomes of many prokaryotes, followed by tedious multiplex-PCR gap filling [10-27]. Generally, these sequencing projects achieved reads of ~ 30-100 folds coverage, assembled into ~30-80 contigs(~4-30 scaffolds). Paired end Roche/454 reads have been sequenced in some of the above reports [17,27]. Roche/454 runs combined with an Illumina/Solexa runs have been implemented in latest reports [10,20-22,24]. Another strategy of single end (shot gun) Roche/454 runs followed by multiplex-PCR gap filling have also been reported in some of the above cases [17,23,25,26]. Is there a cost-effective and universal strategy for the complete prokaryotic genomic sequencing? To answer this question, we carried out a systematic investigation for the assembly qualities with simulated reads generated by Roche/454 platform from 100 randomly selected genomes.

## Materials and methods

### Selection of genomes

100 full-length prokaryotic genomic sequences were randomly selected and downloaded from NCBI Genbank database (Additional file 1: Table S1).

### Generation of simulated 454 shotgun reads and paired end reads

Metasim and Flowsim are both pyrosequencing simulators, which generate pyrosequencing reads in different manners. Metasim is based on parametric model, while Flowsim is based on empirical model which is much more similar to the real data. Metasim can generate both single end reads (SE) and paired end reads (PE), while Flowsim can only generate single end reads. In our simulation studies, Flowsim 0.2.7 [28] was used to generate 454 simulated shotgun reads (100 bp, 200 bp and 400 bp in length). The shotgun reads of 6×, 10×, 15× and 20× redundancy were all generated by the default parameters.

Metasim 0.9.5 [29] was used to generate 454 simulated paired end reads (200 bp in length) from 8 kb insert length library (3 kb/8 kb insert length library for comparison with real *Salmonella Typhimurium *data). Then, recover all the Metasim reads to their original sequences according to their genomic positions. Then, extend each read by 500 bp in length according to their genomic positions. Subsequently, Monte Carlo method [30] based on empirical distribution of Flowsim mutation/indel rate on each site of the reads was used to introduce mutations/indels into all the reads. Finally, Monte Carlo method based on empirical distribution of the read length of the real 454 data was applied to process the above reads to produce the final simulated paired end reads.

### Genome assembly

After combining the simulated paired end and shotgun reads at different depth, we used Newbler 2.5.3 (official assembly software for 454 pyrosequencing reads) [31] to assemble the combined reads into contigs/scaffolds.

### Genome coverage and single base accuracy calculation

MUMmer 3.0 [32] was applied to generate the full-genome alignment between assembly and target genome. The sizes of all alignment blocks were sum up and then divided by the genome length to achieve the final coverage. The alignment block was re-aligned using BLAT [33] with blast format output. Both mutation and indels were counted as variants (mismatches) separately and the final single base accuracy rate would be achieved.

### Gene duplication and loss inference

Gene sets were achieved from Genbank for target 100 genomes and *E. coli K12 *(accession no: AC_000091). All the genes were mapped to the assemblies with BLAT first and refined the alignment with sim4 [34]. Only alignments with identity > 99% and gene length coverage > 0.5 in a single contig will be defined as a gene copy. If one gene cannot be mapped to any of the assembled contigs according to above criteria, the gene would be counted as one false lost gene. If one gene has more duplicates in the assembly than the real value in the target genome, the number difference will be counted as false gene duplicates.

### Sequencing of *E. coli K12 *and a *S. Typhimurium *strain by 454

A single end run (185,587 reads, ~400 bp read length) and a paired end run (199,197 reads, ~400 bp read length, 8 kb paired end library) for *E. coli K12 *strain (accession no: AC_000091) were achieved by 454 GS Jr. Titanium. A single end run (557,464 reads, ~400 bp read length) and a paired end run (558,887 reads, ~350 bp read length, 3 kb paired end library) for a *S. Typhimurium *strain were achieved by 454 FLX Titanium.

### Identification of repeat regions

Repeatscout was used to de novo identify repeat families in each genome [35]. Then RepeatMasker (A.F.A. Smit, R. Hubley & P. Green RepeatMasker at http://repeatmasker.org) was applied to screen repeat sequences in each prokaryotic genome with its own repeat library.

### Statistical methods

R language http://www.r-project.org/ was used for statistical analysis. The linear regression analysis was carried out for 100 genomes under 400 bp/200 bp/100 bp read length sequencing conditions between the genome quality indicators and the repeat content indicators, including number of repeats in the genome, total repeat length of the genome, percentage of the total repeat length of the genome, total repeat length (> 300 bp) of the genome, percentage of the total repeat length (> 300 bp) of the genome, total repeat length (> 700 bp) of the genome and percentage of the total repeat length (> 700 bp) of the genome, respectively.

## Results

### Genome coverage, contigs size and number, and scaffolds number

**Figure 1:**
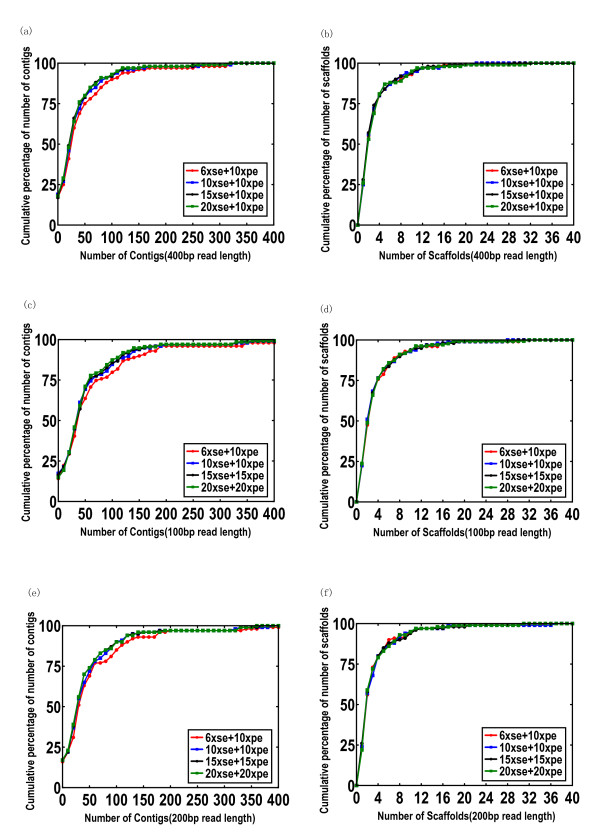
**Number of contigs/scaffolds from assembly results of 100 randomly selected prokaryotic genomes by different sequencing strategies**. (a) Cumulative percentage distribution of number of contigs from 100 genomes by different sequencing strategies. (b) Cumulative percentage distribution of number of scaffolds from 100 genomes by different sequencing strategies.

The genomic sizes of 100 randomly selected genomes range from 100 kb to 8 Mb (Additional file 2: Figure S1a). Over 90% of genomic sizes are less than 5.5 Mb (Additional file 2: Figure S1b). The average genome coverage of assemblies of combined reads of shotgun and paired end are over 98% (Tables 1, 2 and 3) under sequencing depth 16-36× with read length 100 bp, 200 bp and 400 bp. The contig number is < 150 for 95% of 100 genomes (Figure 1a), < 200 for 95% of 100 genomes (Figure 1c) and < 180 for 95% of 100 genomes (Figure 1e). The scaffold number is < 10 for 90% of 100 genomes (Figure 1b), < 12 for 90% of 100 genomes (Figure 1d) and < 11 for 90% of 100 genomes (Figure 1f) with all the combined strategies with sequencing depth 16-36× (400 bp, 100 bp and 200 bp read length). The average numbers of contigs from the above combined sequencing strategies (400 bp, 100 bp and 200 bp read length) range from 51 to 42 (Table 1), 72.1 to 59.7 (Table 2) and 61.1 to 52.9 (Table 3). And the average numbers of scaffolds from the above combined sequencing strategies (400 bp, 100 bp and 200 bp read length) are ~3.5 (Table 1) ~4 (Table 2) and ~3.6 (Table 3). This result implies that longer read length may produce fewer contigs/scaffolds. Subsequently, we checked all the largest scaffolds of simulated 100 genomic assembly results and found that ~90% of the assemblies have a scaffold (the largest one) covering ~98% of their own genomic sequence (data not shown).

**Table 1:** Main average indices in different sequencing strategies for 100 genomes (400 bp read length, 8 kb paired end library)

ST	GCE(%)	SBE(%)	IDR(%)	FLT(%)	FDT(%)	CN	NB	SN
6 × SE + 10 × PE	98.26971	0.004915	0.000364	0.310807	0.4678237	50.94	331136.7	3.64

10 × SE + 10 × PE	98.30248	0.004265	0.000322	0.2626039	0.5629617	44.75	383793.6	3.51

15 × SE + 10 × PE	98.32861	0.003293	0.000294	0.2518801	0.6041274	43.12	397060.7	3.48

20 × SE + 10 × PE	98.35117	0.00227	0.000293	0.2307405	0.6301239	42.3	411169.2	3.66

ST: Sequencing Strategy

SE: Single end reads

PE: paired end reads

GOE: Genome Coverage Rate

SBE: Single Base Error Rate

IDR: Indel Error Rate

FLT: False Gene Duplication Rate

FDT: False Gene Loss Rate

CN: Contig Number

NB: Contig N50 Size(bp)

SN: Scaffold number

**Table 2:** Main average indices in different sequencing strategies for 100 genomes (100 bp read length, 8 kb paired end library)

ST	GCE(%)	SBE(%)	IDR(%)	FLT(%)	FDT(%)	CN	NB	SN
6 × SE + 10 × PE	98.06775	0.00498	0.000339	0.4892094	0.190552	72.11	209661.1	4

10 × SE + 10 × PE	98.09051	0.003982	0.000324	0.4596817	0.180621	63.08	240424.9	3.8367

15 × SE + 10 × PE	98.08065	0.004018	0.000322	0.4731213	0.1733068	61.77	241163.8	3.9184

20 × SE + 10 × PE	98.10211	0.004231	0.000339	0.4754001	0.1754001	59.65	244658.8	3.7642

ST: Sequencing Strategy

SE: Single end reads

PE: paired end reads

GOE: Genome Coverage Rate

SBE: Single Base Error Rate

IDR: Indel Error Rate

FLT: False Gene Duplication Rate

FDT: False Gene Loss Rate

CN: Contig Number

**Table 3:** Main average indices in different sequencing strategies for 100 genomes (200 bp read length, 8 kb paired end library)

ST	GCE(%)	SBE(%)	IDR(%)	FLT(%)	FDT(%)	CN	NB	SN
6 × SE + 10 × PE	98.17144	0.003195	0.000334	0.4401864	0.2416131	61.15	253000.7	3.625

10 × SE + 10 × PE	98.15661	0.004024	0.000317	0.4076573	0.2861061	54.33	290749.3	3.7188

15 × SE + 10 × PE	98.16915	0.004743	0.000305	0.3916122	0.261398	53.47	301038.3	3.64

20 × SE + 10 × PE	98.17177	0.004877	0.000309	0.409125	0.2509012	52.98	289864.6	3.6

ST: Sequencing Strategy

SE: Single end reads

PE: paired end reads

GOE: Genome Coverage Rate

SBE: Single Base Error Rate

IDR: Indel Error Rate

FLT: False Gene Duplication Rate

FDT: False Gene Loss Rate

CN: Contig Number

Contig N50 size is the smallest contig size, in which 50% length of a genome sequences reside in. Our result indicates that the average contig N50 sizes of 6×SE + 10×PE, 10×SE + 10×PE, 15×SE + 10×PE and 20×SE + 10×PE sequencing strategies (400 bp read length) are 331 kb, 384 kb, 397 kb and 411 kb, respectively (Table 1). Contig N50 size of assembly with 400 bp read length is much larger than those of with 100 bp or 200 bp read length (Tables 1, 2 and 3). This result illustrates that longer reads can yield significantly more continuous assemblies for prokaryote genomes.

### Single base accuracy

Single base accuracy (SBA) of an assembly, composed of single base error rate (SBE) and indel error rates (IDR), is one of the most important indicator for evaluating the quality and applicability of the genome assembly. It is generally believed that insertion and deletion (indel) are usually introduced to pyrosequencing reads in regions of homopolymers. Here, we calculate the single SBE and IDR in the assembly results. Our study shows that the average SBA of 6×SE + 10×PE, 10×SE + 10×PE, 15×SE + 10×PE and 20×SE + 10×PE sequencing strategies (400 bp read length) for 100 genomes are all below 0.01% (Table 1). The SBE index doesn't change much compared to the corresponding results from both 100 bp and 200 bp read length for 100 genomes (Tables 1, 2 and 3). Then, we also calculate the IDR in the assembly results. Our result shows that the average IDR from 6×SE + 10×PE, 10×SE + 10×PE, 15×SE + 10×PE and 20×SE + 10×PE sequencing strategies for 100 genomes are all below 0.0005% (Table 1). The IDR index doesn't change significantly compared to the corresponding results from both 100 bp and 200 bp read length for 100 genomes (Tables 1, 2 and 3).

Taken the average SBE and the average IDR together, the average SBA are > 99.99% for prokaryote genome assemblies. This result indicates that the SBA will get saturated as the sequencing depth reaches ~16× sequencing depth.

### False gene duplication/loss rate

Gene duplication, loss, horizontal transfer, and genomic rearrangement are usually involved in the evolutionary processes of bacterial genomes. Mis-assembled regions could mislead these analysis of genomic structure variation into a wrong conclusion. We further calculate the false gene duplication/loss rate and our result in Table 1 reveals that average false gene duplication/loss rates (FDT/FLT) in de novo assembly of different sequencing strategies (400 bp read length) for 100 genomes are 0.47-0.63%/0.23-0.31% for FDT/FLT, respectively. As the read length increases from 100 bp to 400 bp, the FLT decreases slightly and the FDT increases slightly (Tables 1, 2 and 3). This result showed that the FLT/FDT indexes from > 16× next-generation sequencing are applicable for large-scale comparative genomic analysis.

### Influence of genome repeat content on assembly quality

Although it's generally accepted that repeats have significant impact on the assembly result, it is still not clearly demonstrated yet that how repeats can influence the assembly quality indicators with different sequencing strategies. We perform a correlation study between repeats of different length and all the assembly quality indicators under different sequencing strategies for 100 genomes. In Additional file 3: Table S2, the correlation analysis (for 400 bp read length) indicates that: a) significantly positive correlation between total size of long repeats (> 300 bp) and number of contigs (R^2^: range from 0.66 to 0.78; *P *value < 1E-15); b) significantly positive correlation between total size of long repeats (> 700 bp) and number of contigs (R^2^: range from 0.69 to 0.79; *P *value < 1E-15); c) moderately positive correlation between total size of long repeats (> 300 bp) and number of scaffolds (R^2^: range from 0.41 to 0.56; *P *value < 1E-15); d) moderately positive correlation between total size of long repeats (> 700 bp) and number of scaffolds (R^2^: range from 0.44 to 0.58; *P *value < 1E-15). Our result also reveals that the R^2 ^(repeat length vs. number of contigs) decrease as the sequencing read length increases from 100 bp (Additional file 4: Table S3)/200 bp (Additional file 5: Table S4) to 400 bp (Additional file 3: Table S2).

### Reality evaluation of simulated data

**Figure 2:**
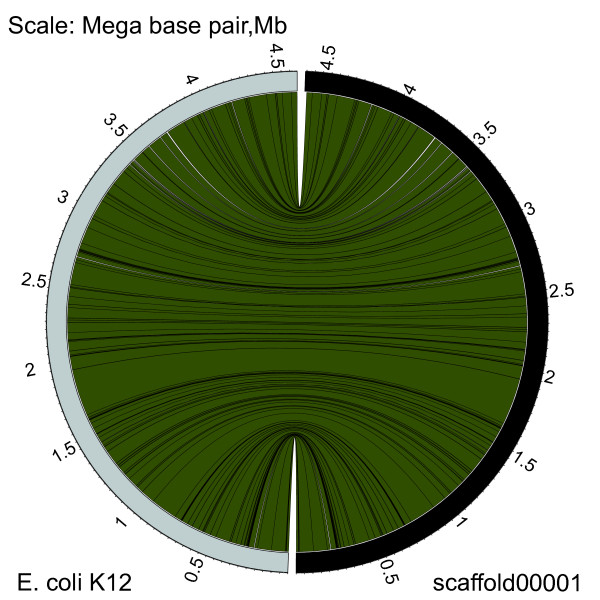
**Alignment results of the full-length genomic sequence of the E. coli K12 strain and the first scaffold from its assembly result by 10×SE + 10×PE sequencing strategy (real data)**. E. coli K12 sequence is on the left side and the sequence of the first scaffold is on the right side. Ribbons represent the alignment blocks extracted from the MUMmer result.

In order to avoid an overestimated evaluation, we investigated the assembly qualities using 6× and 10× machine generated data of an *E. coli K12 strain *(W3110, accession no:AC_000091) from a single end run and a paired end (8 kb paired end library) run of 454 Jr. Our results (Additional file 6: Table S5) show assembly qualities based on real data (qualified run) are very similar with those based on simulation data in every quality indicator evaluated in this study. We further checked the scaffolds assembled by 10×SE + 10×PE sequencing strategy (real data) and found that the largest scaffold from assembly had already covered over 98% of the full-length genomic sequence of the *E. coli K12 strain *(Figure 2). This result means that the rest of our simulation data may be similar to the real data and not overestimated compared to the data of real runs.

We also investigated the assembly qualities using 6×,10×,15× and 20× machine generated data of a *S. Typhimurium *strain from a single end run and a paired end (3 kb paired end library) run of 454 FLX Titanium. Compared to the average indices of simulated data of all 17 *Salmonella *strains from Genbank (accession no: NC_003197, NC_003198, NC_004631, NC_006511, NC_006905, NC_010067, NC_010102, NC_011080, NC_011083, NC_011094, NC_011147, NC_011149, NC_011205, NC_011274, NC_011294, NC_012125 and NC_015761), the contig/scaffold number of real data are similar with those of the average simulated data (Tables 4 and 5). As the sequencing depth increases, the contig/scaffold number only decreases a little. For comparison, we simulated a same data set of all 17 *Salmonella *strains from Genbank by a single end run and a paired end run (8 kb paired end library) of 454 FLX Titanium. Interestingly, the contig/scaffold number of the simulated data significantly decreased (Tables 4 and 6). Then, we further check all the largest scaffolds assembled by 10×SE + 10×PE sequencing strategy (8 kb paired end library) for each simulated data and found that the largest scaffolds have already covered over 98% of their full-length genomic sequence. This result does agree with that of the *E. coli K12 *strain and imply that 8 kb paired end library made a significant difference rather than 3 kb paired end library.

**Table 4:** Main average indices in different sequencing strategies for 17 *Salmonella *genomes (400 bp read length, 3 kb paired end library)

ST	GCE	SBA	IDR	FLT	FDT	CN	NB	SN
6 × SE + 10 × PE	98.77307189	0.000523727	0.000125695	0	0.020605811	62	219592	17

10 × SE + 10 × PE	98.79345172	0.000439829	4.19E-05	0	0.020605811	51	224172	16

15 × SE + 10 × PE	98.71993949	0.001487248	4.19E-05	0	0.061817433	48	224247	15

20 × SE + 10 × PE	98.70417356	0.000796407	2.10E-05	0	0.061817433	48	224241	16

ST: Sequencing Strategy

SE: single end 454 FLX Titanium reads

PE: paired end 454 FLX Titanium reads

GCE: genome coverage rate of the assembly result

SBA: single base accuracy rate of the assembly result

IDR: indel rate of the assembly result

FDT: false gene duplication rate of the assembly result

CN: large contig number

NB: N50 bp length of the assembly result

SN: scaffold number

**Table 5:** Main average indices in different sequencing strategies for read data of a *S. Typhimurium *strain (400 bp read length, 3 kb paired end library)

ST	CN	NB	SN
6 × SE + 10 × PE	85	149382	17

10 × SE + 10 × PE	77	195805	17

15 × SE + 10 × PE	77	195806	19

20 × SE + 10 × PE	81	195811	17

all SE + PE	73	196924	15

ST: Sequencing Strategy

SE: single end 454 FLX Titanium reads

PE: paired end 454 FLX Titanium reads

CN: large contig number

NB: N50 bp length of the assembly result

SN: scaffold number

**Table 6:** Main average indices in different sequencing strategies for 17 *Salmonella *genomes (400 bp read length, 8 kb paired end library)

ST	GCE	SBA	IDR	FLT	FDT	CN	NB	SN
6 × SE + 10 × PE	98.71211859	0.000565917	0.000188639	0	0.020605811	58	201619	5

10 × SE + 10 × PE	98.80971421	0.000251291	2.09E-05	0	0.020605811	51	224241	5

15 × SE + 10 × PE	98.70601499	0.000796358	2.10E-05	0	0.061817433	47	224247	6

20 × SE + 10 × PE	98.70003553	0.000859158	2.10E-05	0	0.103029054	46	219591	5

ST: Sequencing Strategy

SE: single end 454 FLX Titanium reads

PE: paired end 454 FLX Titanium reads

GCE: genome coverage rate of the assembly result

SBA: single base accuracy rate of the assembly result

IDR: indel rate of the assembly result

FDT: false gene duplication rate of the assembly result

CN: large contig number

NB: N50 bp length of the assembly result

SN: scaffold number

## Discussion

Complete prokaryotic genome is the basis for high-resolution comparative genomic analysis. Lots of complete prokaryotic genomes have been sequenced in the last decade, but the Genbank database deposits only some of them. Finishing a genome also includes tedious gap filling. If there are too many (like hundreds) gaps in the draft assembly, the task of gap filling will be quite exhausting and time-consuming.

All simulated reads in this study are generated according to the empirical model of real 454 runs. Thus, compared to the real data (*E. coli K12*, accession no: AC_000091) from the 454 Jr. run, our result shows that the simulated data (both the distribution of mutation/indel rate on each site of the reads and the distribution of the read length) are very similar to the real data (data not shown). Things are similar when we compared the real data and the simulated data from a *Salmonella *strain in terms of contig/scaffold number. Another interesting result is that the number of scaffold significantly decreases from 16 to 5 (10×SE + 10×PE, Tables 4 and 5) as the paired end library fragment increases from 3 kb to 8 kb in *Salmonella*'s result. Lacking of the full genome sequence of this *Salmonella *strain, we can't compare the other indices between the simulated data and the real data.

Contigs can be oriented into scaffolds with the paired end information, but the traditional fosmid paired end library construction is time-consuming and expensive. Larger insertion size of the paired end library could bridge longer gaps and yield overall more continuous assembly [36,37]. According to a previous study on *E. coli K12 *genomic sequencing [37], 3 kb paired end 454 library (250 bp read length) was capable of bridging the 0.2 kb-1.5 kb gaps between the contigs. Nowadays, the 8 kb paired end 454 library (400 bp read length), proposed by Roche/454, is technically mature and believed to bridge longer gaps in the application of bacterial genomic sequencing. Moreover, it only took scientists several days to construct such a paired end library. Thus, this kind of paired end library could be an alternative solution to the expensive traditional fosmid paired end library.

Based on this simulation study, an effective sequencing strategy is proposed to achieve the complete prokaryotic genome for most of prokaryotic genomes by a single end 454 Jr. run (400 bp read length, Titanium) and a paired end 454 Jr. run (8 kb library, 400 bp read length, Titanium), respectively followed by multiplex-PCR gap filling (for ~ < 10 scaffolds) for the gaps between scaffolds. It is easy to fill the gaps in each scaffold by PCR, because the sequences flanking each gap are very clear to facilitate the primer design. Multiplex-PCR will be applied to close the gaps between scaffolds, because of the unavailability of scaffolds' orders and orietations. Combinations of different primers, designed according the scaffolds' ends, will be mixed in each PCR reaction. The more combinations, the more multiplex-PCR reactions there will be. However, if there are only ~ < 10 scaffolds, the multiplex-PCR reactions will be limited and easy to do. Generally, the above strategy is able to finish a draft prokaryotic genome within days. It also facilitates the process of multiplex-PCR gap filling by knowing the order/orientation of most contigs.

Our result suggests that with a combination of one run of single end and one run of paired end reads, 90% of the 100 genome assemblies are less than 10 scaffolds and 95% of 100 genomes assemblies are less than 150 contigs (400 bp read length). Despite of some extreme genomes like NC_013361 (327 contigs/30 scaffolds) containing high proportion (> 10% of the genome) of long repeats (> 300 bp), most of the selected genomes can be assembled into high quality draft genomes (< 50 contigs, ~4 scaffolds, > 330 kb contig N50 size, > 99.99% single base accuracy and < 0.7% false gene duplication/loss rate). The correlation analysis reveals the high correlation between total size of long repeats and the number of contigs. It suggests that the fragmented draft assemblies are caused by long repeats which agrees with our previous finding [38]. The correlation analysis also reveals the correlation coefficient (between total size of repeat length and the number of contigs) decreases as the read length increases, which proposes that problems caused by repeats could be solved by increasing the read length in the 454 run.

Given all the assembly quality indicators are saturated at 10×SE + 10×PE/15×SE + 10×PE sequencing strategy (400 bp read length) and over 90% of all randomly selected genomic sizes are less than 5.5 Mb, 454 Jr. is the best choice of all the pyrosequencing technology in terms of producing enough sequencing depth (average ~10x-15x for most prokaryotic genomes) and relatively longer reads (400 bp read length) at a lower cost.

In summary, we propose a both cost-effective and universal strategy for the complete prokaryotic genomic sequencing based on computer simulation and further confirmed by two sets of real data analysis (*E. coli K12 *strain and a *S. Typhimurium *strain). This strategy may help sequencing most of the complete genomes within a much shorter period and thus probably open the door to large-scale complete genome sequencing.

## Competing interests

The authors declare that they have no competing interests.

## Authors' contributions

FCL and JJ designed this study. JJ and JL designed and carried out all the analysis. HSK, CHA, PTWL, LL, KMK and JMLL carried out the *S. Typhimurium *genome sequencing. JJ, JL and FCL wrote the manuscript. All authors read and approved the final manuscript.

## Supplementary Material

Additional file 1 **Table S1**. Basic information of 100 selected prokaryotic genomes. Click here for file

Additional file 2 **Figure S1**. Genome sizes of 100 randomly selected prokaryotic genomes. (**a**) Percentage distribution of 100 genome sizes. (**b**) Cumulative percentage distribution of 100 genome sizes. Click here for file

Additional file 3 **Table S2**. Linear regression results for 100 genomes under 400 bp read length sequencing condition between the genome quality indicators and the number of repeats in the genome/the total repeat length of the genome/the percentage of the total repeat length of the genome/the total repeat length (> 300 bp) of the genome/the percentage of the total repeat length (> 300 bp) of the genome/the total repeat length (> 700 bp) of the genome/the percentage of the total repeat length (> 700 bp) of the genome. Click here for file

Additional file 4 **Table S3**. Linear regression results for 100 genomes under 100 bp read length sequencing condition between the genome quality indicators and the number of repeats in the genome/the total repeat length of the genome/the percentage of the total repeat length of the genome/the total repeat length (> 300 bp) of the genome/the percentage of the total repeat length (> 300 bp) of the genome/the total repeat length (> 700 bp) of the genome/the percentage of the total repeat length (> 700 bp) of the genome. Click here for file

Additional file 5 **Table S4**. Linear regression results for 100 genomes under 200 bp read length sequencing condition between the genome quality indicators and the number of repeats in the genome/the total repeat length of the genome/the percentage of the total repeat length of the genome/the total repeat length (> 300 bp) of the genome/the percentage of the total repeat length (> 300 bp) of the genome/the total repeat length (> 700 bp) of the genome/the percentage of the total repeat length (> 700 bp) of the genome. Click here for file

Additional file 6 **Table S5**. Comparison of the genome quality indicators between real data and simulated data under 6×SE + 10×PE and 10×SE + 10×PE sequencing strategies. Click here for file
