# Supplementary figures and images for: A cost-effective and universal strategy for complete prokaryotic genomic sequencing proposed by computer simulation
Source: BMC Res Notes. 2012 Jan 31;5:80. doi: 10.1186/1756-0500-5-80 (PMC3296665; doi:10.1186/1756-0500-5-80)

(a)

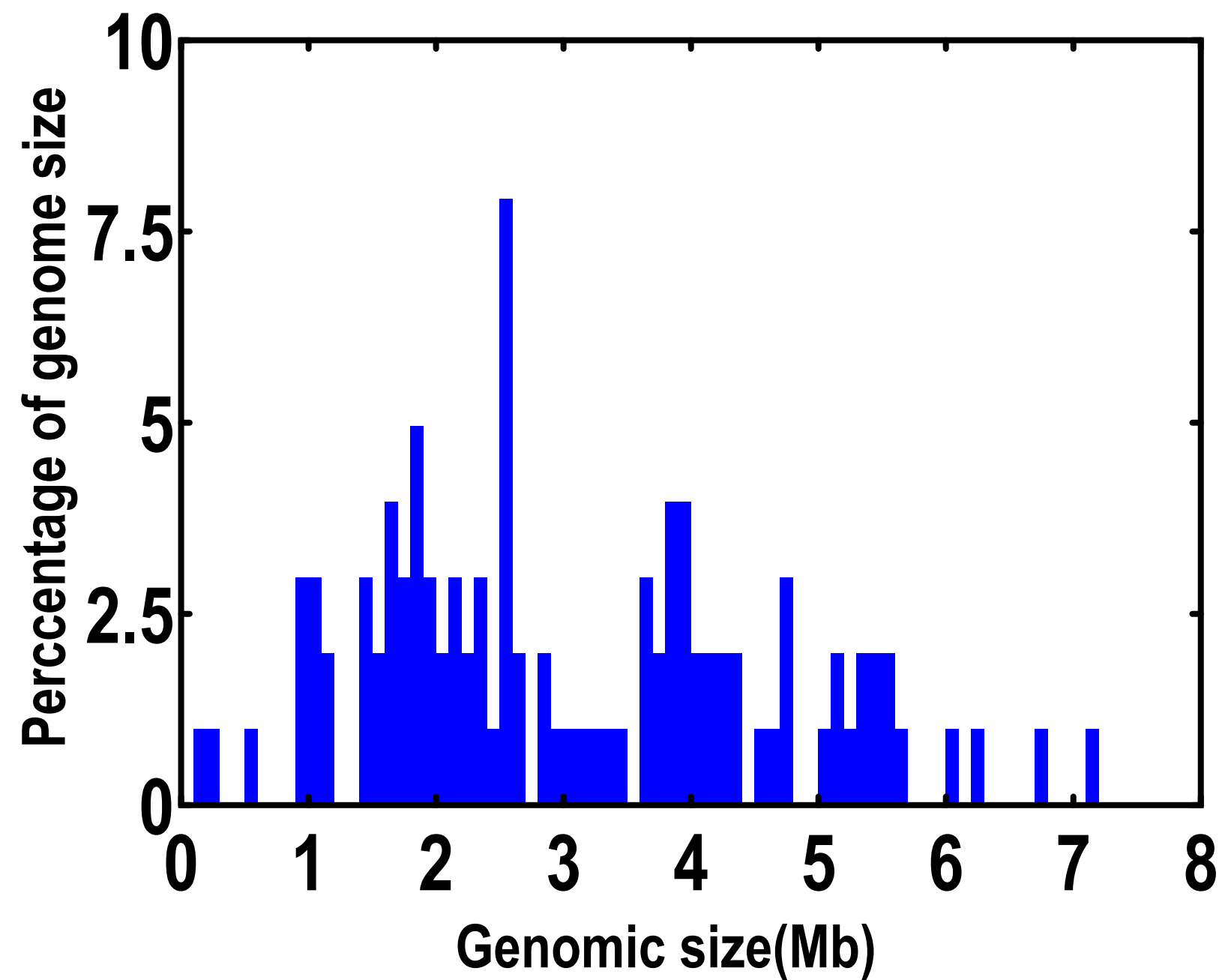

(b)

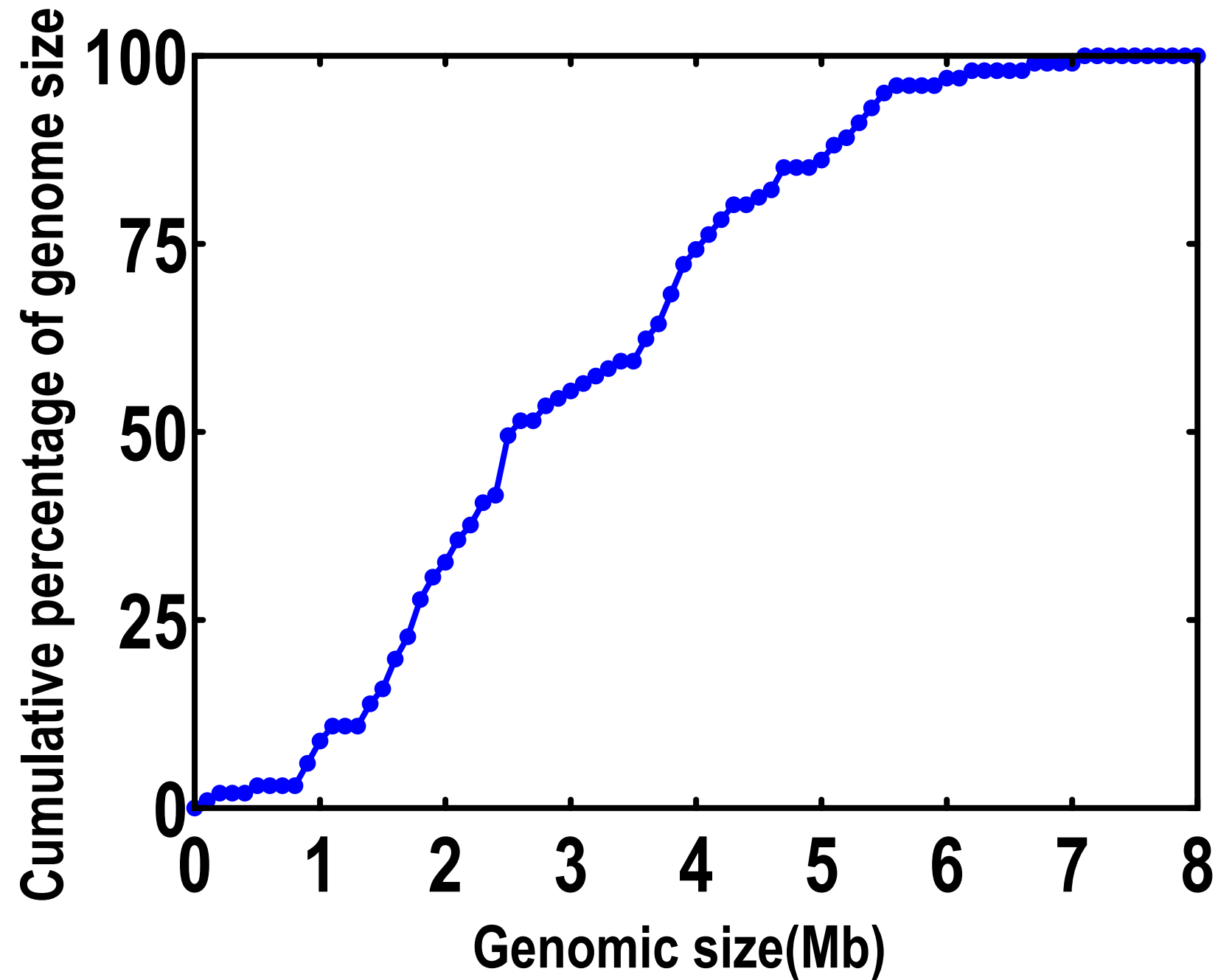

Supplement: Additional file 2 — Figure S1. Genome sizes of 100 randomly selected prokaryotic genomes. (a) Percentage distribution of 100 genome sizes. (b) Cumulative percentage distribution of 100 genome sizes. [file 1756-0500-5-80-S2.PDF]
